# Supplementary material for: Chromatin-Directed Proteomics Identifies ZNF84 as a p53-Independent Regulator of p21 in Genotoxic Stress Response
Source: Cancers (Basel). 2021 Apr 27;13(9):2115. doi: 10.3390/cancers13092115 (PMC8123910; doi:10.3390/cancers13092115)
Supplement: Supplementary file 1 [file cancers-13-02115-s001.zip › cancers-1169411-supplementary.pdf]

cancers

MDPI

Article

Chromatin-Directed Proteomics Identifies ZNF84 as a p53-Independent Regulator of p21 in Genotoxic Stress Response

Anna Strzeszewska-Potyrała [1,*], Karolina Staniak [1], Joanna Czarnecka-Herok [1], Mahmoud-Reza Rafiee [2], Marcin Herok [1], Grażyna Mosieniak [1], Jeroen Krijgsveld [3,4] and Ewa Sikora [1]

[1] Laboratory of Molecular Bases of Aging, Nencki Institute of Experimental Biology, PAS, 3 Pasteur Street, 02-093 Warsaw, Poland; k.kucharewicz@nencki.edu.pl (K.S.); j.czarnecka@nencki.edu.pl (J.C.-H.); marcin.herok@gmail.com (M.H.); g.mosieniak@nencki.edu.pl (G.M.); e.sikora@nencki.edu.pl (E.S.)

[2] Bioinformatics and Computational Biology Laboratory the Francis Crick Institute, 1 Midland Road, London NW1 1AT, UK; mahmoud-reza.rafiee@crick.ac.uk

[3] German Cancer Research Center (DKFZ), Division of Proteomics of Stem Cells and Cancer, Im Neuenheimer Feld 581, 69120 Heidelberg, Germany; j.krijgsveld@dkfz-heidelberg.de

[4] Medical Faculty, Heidelberg University, Im Neuenheimer Feld 672, 69120 Heidelberg, Germany

* Correspondence: a.strzeszewska@nencki.edu.pl

Supplementary

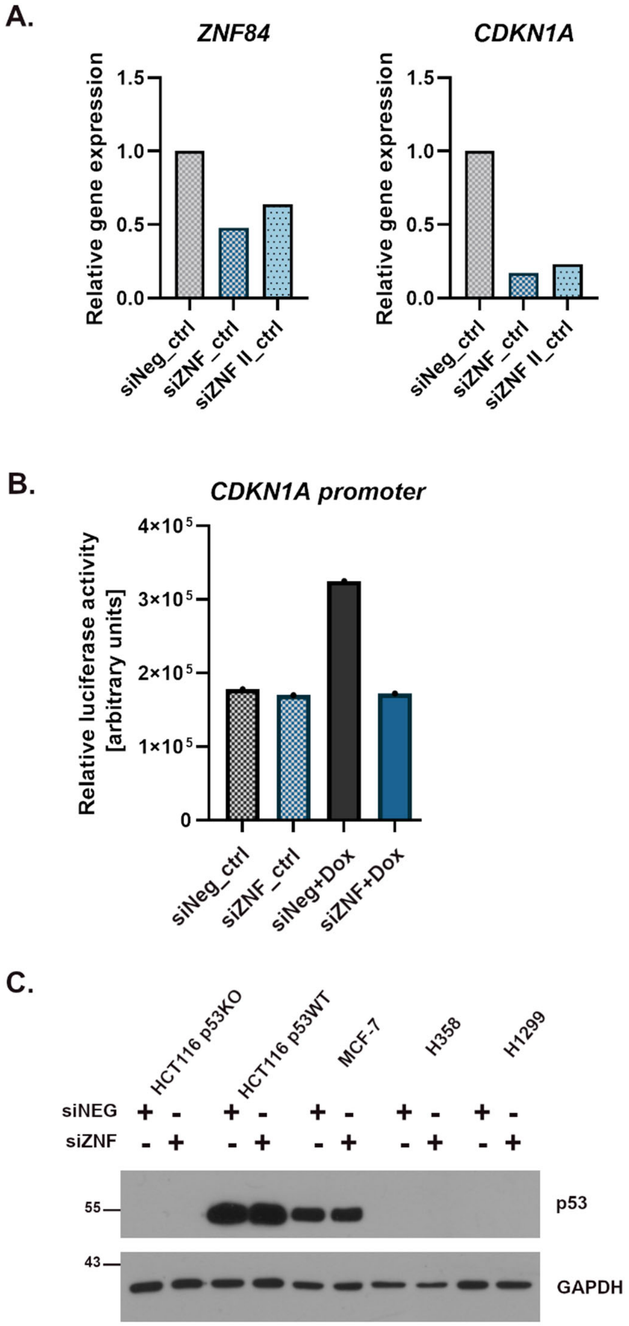

Figure S1. Additional information on the experimental model used in the study. (**A**) RT-PCR analysis of *ZNF84* (graph on the left) and *CDKN1A* (graph on the right) mRNA in HCT116 p53KO cells upon transfection with control siRNA (siNeg, grey bars) or ZNF84-targeting siRNA: siZNF (checked dark blue bars) or siZNF_II (light blue bars). The fold gene expression values, which are plotted in the graphs, were calculated as 2 to the power of negative ΔΔCt (ΔCt was calculated as the mean of raw Ct values of GAPDH subtracted from the mean Ct of *CDKN1A* or *ZNF84* expression and ΔΔCt was calculated as a difference between ΔCt for ZNF84-targeting sequence and ΔCt for siNeg-transfected cells.); (**B**) Measurement of *CDKN1A* promoter activity by luciferase assay in non-treated (patterned bars) and Dox-treated (solid colour bars) cells. Cells were transfected with either control (indicated by gray colour) or ZNF84-targeting (blue) siRNA. The luminescence values were normalized to the total protein content; (**C**) Western blotting analysis of the total p53

protein level in non-treated siRNA-transfected cells (HCT116 p53KO, HCT116 p53WT, MCF-7, H358, H1299). GAPDH was used as a loading control.

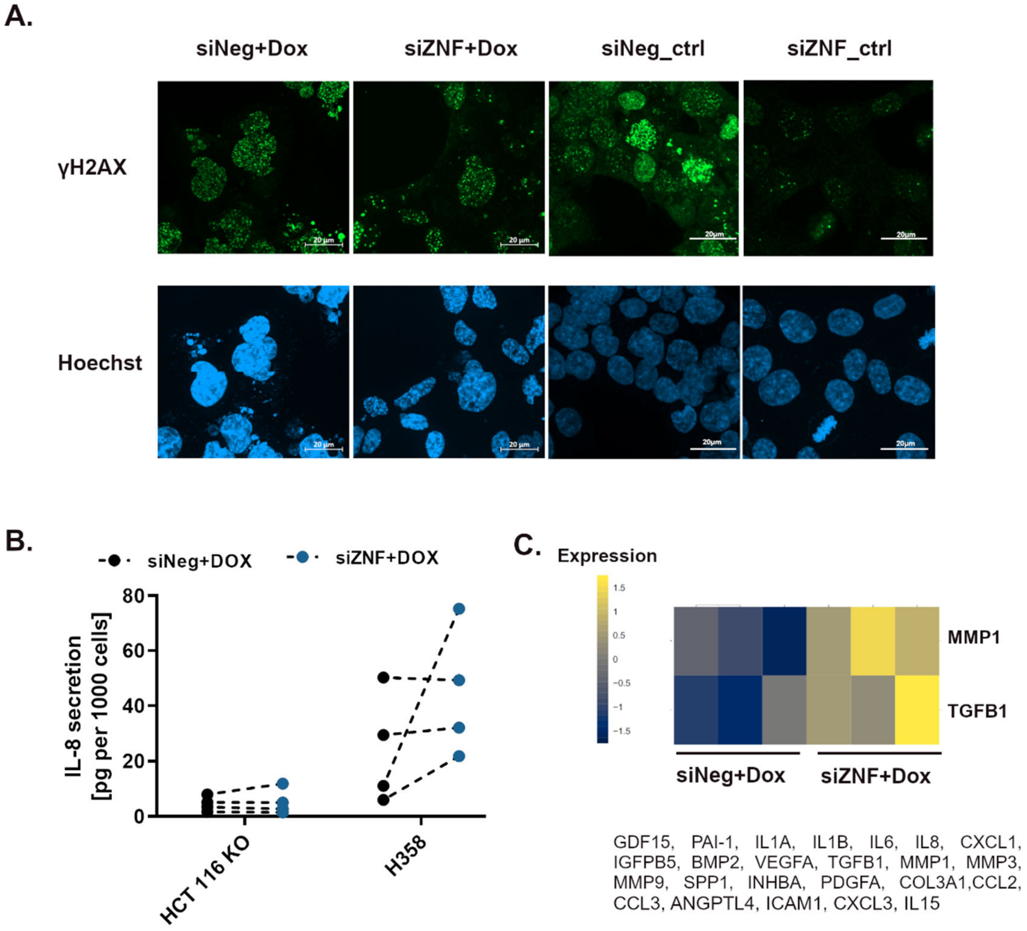

Figure S2. Analysis of the impact of ZNF84 knock-down on senescence. (**A**) Representative images of anti-γH2AX immunofluorescent staining (maximal projection) of HCT116 p53KO cells transfected with control (siNeg) or anti-ZNF84 (siZNF) siRNA and either treated with doxorubicin (Dox) or non-treated (ctrl). Hoechst staining shown below. Scale bars indicating 20 μm in the bottom-right corner of each image; Magnification 630×; (**B**) ELISA measurement of IL-8 in the culture medium of cells treated constantly for 5 days with doxorubicin (HCT116 p53KO on left side of the graph and H358 on the right). Black dots represent results for siNeg-transfected cells, blue - siZNF. The data points for different treatments (siRNAs) from each of the four independent expriments are linked with dashed line; C.Heatmap visualising changes in expression of common SASP genes (names of the analyzed genes indicated under the plot). Gene names in rows, data from individual experiments in columns (first three columns to the left present data from cells transfected with siZNF and treated with Dox for 2 days, then data from siZNF-transfected cells treated in the same way). Colour scale represents log-transformed count values normalized for differences in sequencing depth and composition bias between the samples; adj. p-value<0.05.

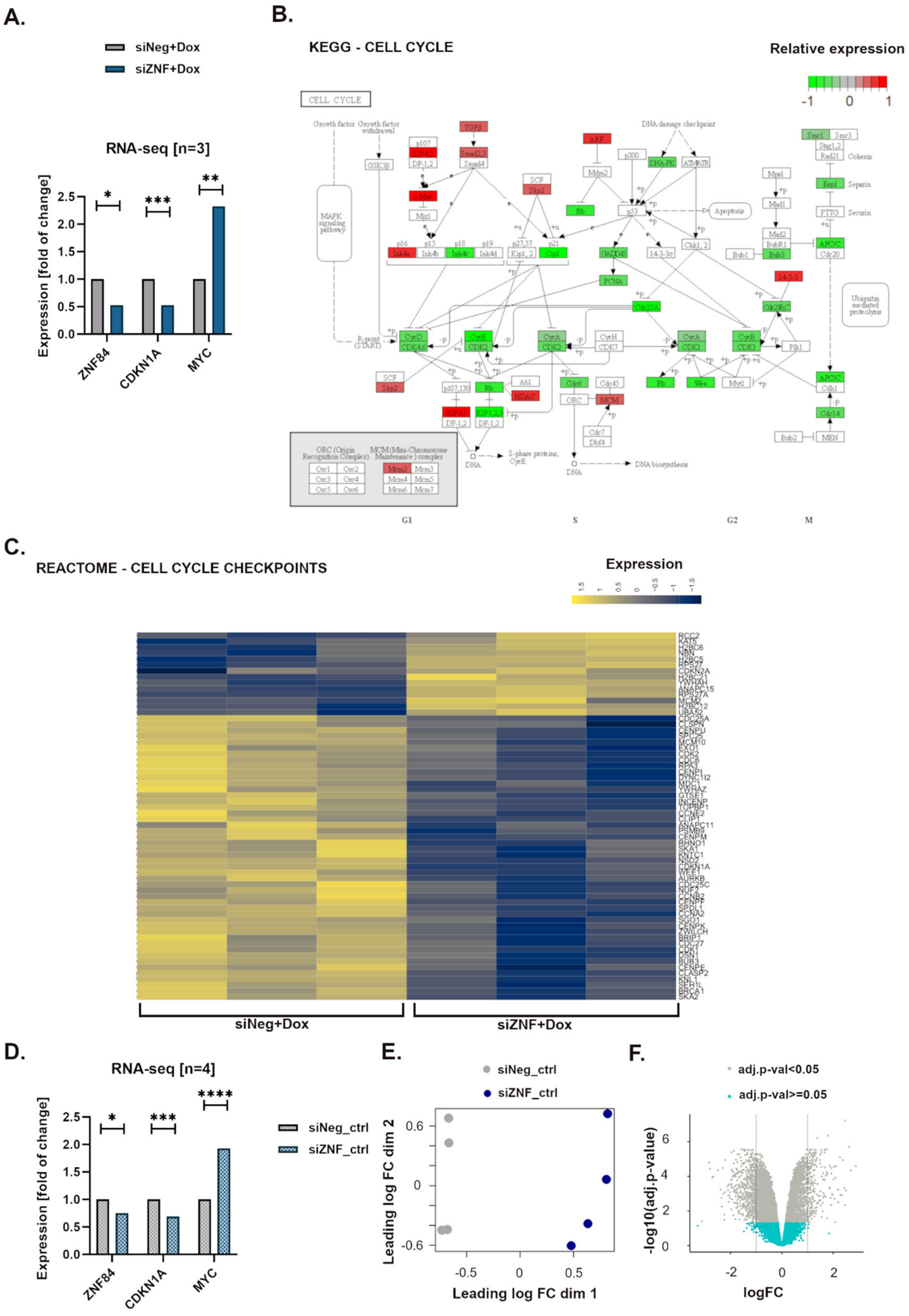

Figure S3. Changes in expression of cell-cycle related genes in ZNF84-deficient doxorubicin-treated HCT116 p53KO cells and expression data from RNA-seq analysis on non-treated cells. (**A**) RNA-seq analysis of *ZNF84, CDKN1A* and *MYC* expression in doxorubicin-treated HCT116 p53KO cells upon transfection with control siRNA (siNeg, grey bars) or ZNF84-targeting siRNA (siZNF, blue bars); (**B**) The KEGG Cell Cycle (hsa04110) pathway graph with gene expression data (Log2 fold changes of gene expression siNeg+Dox vs. siZNF+Dox) coded to a range from -1 to 1 and rendered as pseudo color in Pathview. Only the significally differentially expressed genes (adj. P-value<0.05) are marked in colour; (**C**) Heatmap visualizing changes in expression of genes associated with the activity of cell cycle checkpoints, as defined in REACTOME

database. Gene names in rows, data from individual experiments in columns (left columns represent data from cells transfected with siNeg and treated with Dox for 2 days, right rows siZNF-transfected cells treated in the same way). Colour scale represents log-transformed count values normalized for differences in sequencing depth and composition bias between the samples; adj. P-value<0.05. (**D**) RNA-seq analysis of *ZNF84, CDKN1A* and *MYC* expression in HCT116 p53KO cells upon transfection with control siRNA (siNeg, grey bars) or ZNF84-targeting siRNA (siZNF, blue bars), non-treated with a drug; * indicates adj. *p*-value < 0.05, *** adj. *p*-value < 0.001, **** adj. *p*-value < 0.0001; (**E**) Visualization of a principle components analysis (PCA), which determines the greatest sources of variation in the RNA-seq data. Each point represents an individual sample (non-treated HCT116 p53KO cells, transfected with either control siRNA (gray) or anti-ZNF84 siRNA (blue); 4 independent experiments); (**F**). Volcano plot representing differential expression of genes between siNeg_ctrl and siZNF_ctrl samples (*x*-axis: logarithm of fold change, *y*-axis: negative decimal logarithm of FDR-controlled *p*-value); significantly differentially expressed genes (adj. *p*-value < 0.05) represented by gray points;

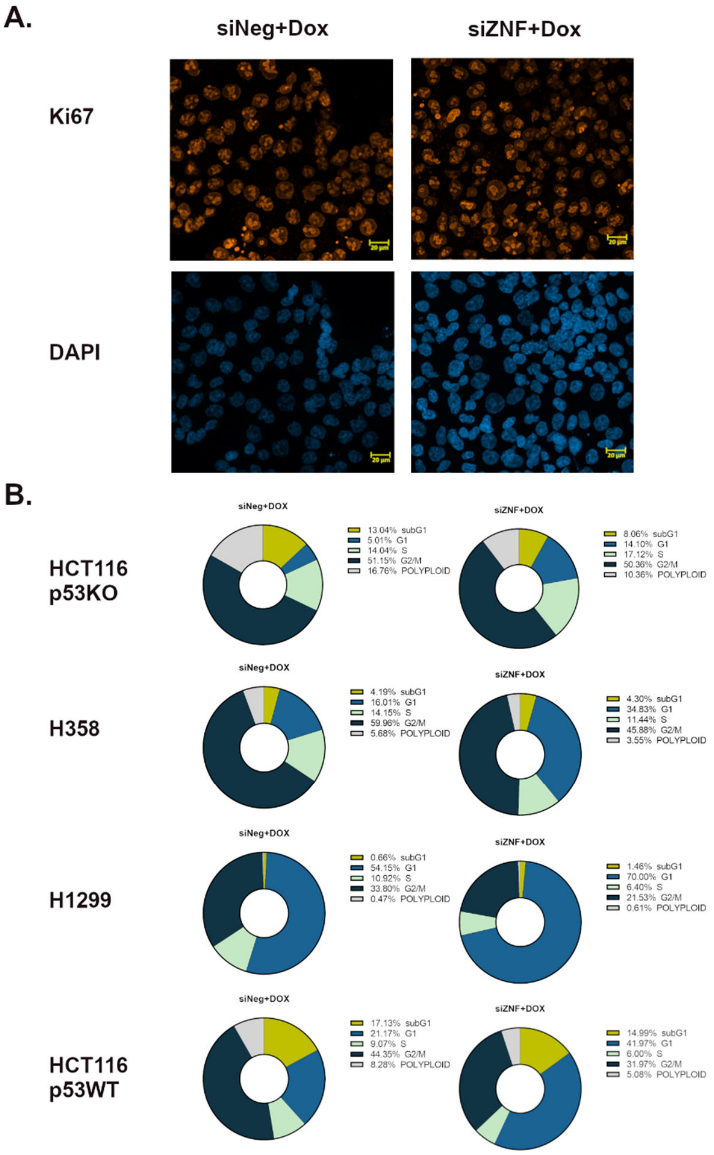

Figure S4. Cell cycle phase deregulation in ZNF84-deficient cells treated with doxorubicin. (**A**) Immunofluorescent detection of Ki67 in HCT116 p53KO cells. Cell nuclei visulaised with DAPI. Magnification 630×; (**B)** Cell cycle phases distribution based on flow-cytometric analysis of PI-stained HCT116 p53KO, H358, H1299 and HCT116 p53WT cells, transfected with either control siRNA (left panel) or ZNF84 siRNA (right panel) and treated with doxorubicin for 24hours. Mean values of percentage of cells in different phases of from at least three independent experiments.

Table S1. Results of Gene Set Enrichment Analysis of the differentially expressed (siNeg vs. siZNF) genes assigned to REACTOME gene sets (selected categories) in control and dox-treated cells.

GENE SET CATEGORY (REACTOME)	UNTREATED SAMPLES		+ DOX SAMPLES	
	P-value	NES	P-value	NES
**Cell cycle**				
RESOLUTION_OF_SISTER_CHROMATID_COHESION	0.001801802	-2.104248	0.002032520	-2.076083
MITOTIC PROMETAPHASE	0.001745201	-2.182460	0.002061856	-2.023935
MITOTIC SPINDLE CHECKPOINT	0.001828154	-1.927063	0.002012072	-1.878157
AURKA ACTIVATION BY TPX2	0.001937984	-1.943927	0.002079002	-1.682434
CELL CYCLE	0.001615509	-1.895465	0.001964637	-1.659310
REGULATION OF PLK1 ACTIVITY AT G2 M TRANSITION	0.001818182	-1.923370	0.004149378	-1.603339
CELL CYCLE CHECKPOINTS	0.001650165	-1.655431	0.001984127	-1.592223
MITOTIC METAPHASE AND ANAPHASE			0.002066116	-1.673285
SIGNALLING TO RAS			0.006072874	-1.857790
SIGNALLING TO ERKS			0.005928854	-1.800462
**Cell death**				
CELL DEATH SIGNALLING VIA NRAGE NRIF AND NADE	0.001883239	-1.749265	0.006097561	-1.609316
NRAGE SIGNALS DEATH THROUGH JNK			0.004149378	-1.755035
APOPTOTIC CLEAVAGE OF CELLULAR PROTEINS			0.007889546	-1.721522
**Translation**				
EUKARYOTIC TRANSLATION INITIATION	0.002222222	3.045449	0.001945525	3.4085626
RRNA PROCESSING	0.002304147	3.053526	0.001945525	3.266821
TRANSLATION	0.002457002	2.843162	0.001984127	3.1672431
MITOCHONDRIAL TRANSLATION	0.002123142	2.231724	0.001945525	2.3033293
POSITIVE EPIGENETIC REGULATION OF RRNA EXPRESSION			0.005882353	1.595668
TRANSPORT OF MATURE TRANSCRIPT TO CYTOPLASM			0.001937984	1.763215
**Oxidative phosphorylation**				
RESPIRATORY ELECTRON TRANSPORT ATP SYNTHESIS BY CHEMIOSMOTIC COUPLING AND HEAT PRODUCTION BY UNCOUPLING PROTEINS	0.002192982	2.302576	0.001941748	2.226829
RESPIRATORY ELECTRON TRANSPORT	0.002173913	2.323937	0.001968504	2.103013
THE CITRIC ACID TCA CYCLE AND RESPIRATORY ELECTRON TRANSPORT	0.002277904	2.282628	0.001919386	2.094223
FORMATION OF ATP BY CHEMIOSMOTIC COUPLING			0.001992032	1.936532
**Mitophagy and mitochondria biogenesis**				
COMPLEX I BIOGENESIS	0.002114165	2.041329	0.001919386	1.9204224
MITOCHONDRIAL BIOGENESIS			0.001926782	1.698715
MITOPHAGY			0.002028398	2.056372
CRISTAE FORMATION			0.002020202	2.051129
PINK PARKIN MEDIATED MITOPHAGY			0.002032520	1.953346
**Response to stress/stress adaptation**				
CELLULAR RESPONSE TO HEAT STRESS	0.002141328	1.655562	0.003891051	1.626925
FORMATION OF TC NER PRE INCISION COMPLEX			0.003846154	1.671051
